# Retinal Vascular Physiology Biomarkers in a 5XFAD Mouse Model of Alzheimer’s Disease

**DOI:** 10.3390/cells11152413

**Published:** 2022-08-04

**Authors:** Nathanael Matei, Sophie Leahy, Norman P. Blair, James Burford, Mansour Rahimi, Mahnaz Shahidi

**Affiliations:** 1 Department of Ophthalmology, University of Southern California, Los Angeles, CA 90033, USA; 2 Department of Ophthalmology and Visual Sciences, University of Illinois at Chicago, Chicago, IL 60612, USA

**Keywords:** Alzheimer’s disease, retinal vascular physiology biomarkers, 5XFAD, amyloid β

## Abstract

Background: Alzheimer’s disease (AD) is a neurodegenerative disorder that affects the brain and retina and lacks reliable biomarkers for early diagnosis. As amyloid beta (Aβ) manifestations emerge prior to clinical symptoms and plaques of amyloid may cause vascular damage, identification of retinal vascular biomarkers may improve knowledge of AD pathophysiology and potentially serve as therapeutic targets. The purpose of the current study was to test the hypothesis that retinal hemodynamic and oxygen metrics are altered in 5XFAD mice. Methods: Thirty-two male mice were evaluated at 3 months of age: sixteen 5XFAD transgenic and sixteen wild-type mice. Spectral-domain optical coherence tomography, vascular oxygen tension, and blood flow imaging were performed in one eye of each mouse. After imaging, the imaged and fellow retinal tissues were submitted for histological sectioning and amyloid protein analysis, respectively. Protein analysis was also performed on the brain tissues. Results: Retinal physiological changes in venous diameter and blood velocity, arterial and venous oxygen contents, coupled with anatomical alterations in the thickness of retinal cell layers were detected in 5XFAD mice. Moreover, an increase in Aβ42 levels in both the retina and brain tissues was observed in 5XFAD mice. Significant changes in retinal oxygen delivery, metabolism, or extraction fraction were not detected. Based on compiled data from both groups, arterial oxygen content was inversely related to venous blood velocity and nerve fiber/ganglion cell layer thickness. Conclusions: Concurrent alterations in retinal hemodynamic and oxygen metrics, thickness, and tissue Aβ42 protein levels in 5XFAD mice at 3 months of age corresponded to previously reported findings in human AD. Overall, these results suggest that this mouse model can be utilized for studying pathophysiology of AD and evaluating potential therapies.

## 1. Background

As life expectancy continues to increase, the prevalence of dementia has grown to ~47 million people worldwide in 2015 [1]. Alzheimer’s disease (AD), the most common type of dementia, is a neurodegenerative disorder that affects the brain and retina, and it lacks effective treatments as well as reliable biomarkers for early diagnosis [2]. As an extension of the diencephalon, the retina displays many pathological characteristics of the Alzheimer brain, e.g., accumulation of amyloid beta (Aβ) oligomers, increased p-Tau, and loss of function [3,4]. To date, diagnosis is based on clinical and neuropsychological examinations in combination with neuroimaging studies, but the abnormalities revealed by these methods occur late in the disease and limit interventions. Therefore, retinal biomarkers may provide therapeutic targets to intervene early and attenuate or even prevent the pathophysiologic process that progresses to symptomatic dementia.

The five-familial AD (5XFAD) transgenic mouse model closely mirrors human AD pathophysiology as early as 2 months of age with clear neuronal degeneration in the brain [5]. Aβ42 levels are specifically increased in 5XFAD mice. In the retina of 5XFAD mice, studies have reported a marked increase in Aβ42/Aβ40 [6,7] and pattern electroretinography impairments [8] at 3 months of age, followed by cognitive impairments as early as 4 months of age [5]. Furthermore, histological evaluation of the retina in a 5XFAD mouse model at 12 months has reported thickening of the Bruch’s membrane and thinning of the retinal pigment epithelium [9]. Using optical coherence tomography (OCT), Lim et al. reported retinal nerve fiber layer (RNFL) thinning at 6 months of age in the 5XFAD mouse model [10]. However, cellular apoptosis was detected in the hippocampus of 5XFAD mice as early as 4 months [11].

As future treatments for AD become available, early-stage diagnosis and timely initiation of the treatments may attenuate or even prevent unfavorable outcomes. Given that Aβ and tau manifestations emerge ~20 years before clinical symptoms [12] and that plaques of amyloid may cause vascular damage, retinal vascular physiological biomarkers may improve AD diagnosis prior to significant neurodegeneration. Increased oxygen saturation in retinal arterioles and venules was shown in AD patients with mild cognitive impairment, suggesting vascular abnormalities to be present with degeneration of retinal cell layers [13,14]; this suggests the potential of oxygen saturation as an ocular biomarker of AD. Moreover, reduced retinal venous blood velocity [15,16,17] and blood flow [15,18] due to the narrowing of the lumen as a result of Aβ deposition in the vessel walls have been reported in patients with early AD. Studies have also documented retinal anatomical changes, namely retinal ganglion cell loss in foveal and parafoveal retinas from postmortem AD patients [19,20].

Optimally, a comprehensive assessment of retinal physiological biomarkers in AD would include hemodynamic metrics (vessel diameter, blood velocity, and blood flow) and oxygen metrics [vascular oxygen contents, oxygen metabolism (MO_2_), delivery (DO_2_), and extraction fraction (OEF)]. As abnormalities in blood flow and oxygen metrics have been shown in human AD, retinal physiological biomarkers in animal models of AD have the potential to improve knowledge of AD pathophysiology. Such knowledge may enable predicting irreversible neurodegeneration and identify a window of opportunity for intervention. The purpose of the current study was to test the hypothesis that retinal hemodynamic and oxygen metrics are altered in 5XFAD mice.

## 2. Materials and Methods

### 2.1. Animals

All experiments were approved by the University of Southern California Institutional Animal Care and Use Committee (IACUC: 20785). The experiments complied with the guidelines of the statement from Use of Animals in Ophthalmic and Vision research by the Association for Research in Vision and Ophthalmology, and they were reported according to the Animal Research: Reporting of In Vivo Experiments guidelines. Thirty-two mice were evaluated. Sixteen male wild-type mice (weight = 26.4 ± 1.7 g; age = 3 months) were obtained from Jackson Laboratory (C57BL/6J; *N* = 10) or Charles River Laboratories (C57BL/6; *N* = 6). Sixteen male 5XFAD transgenic mice (the MMRRC stock 24848, Stock Number: 034848-JAX, and Citation ID: RRID:MMRRC_034848-JAX) (weight = 26.5 ± 2.6 g; age = 3 months) were obtained from the Jackson Laboratory (Bar Harbor, ME, USA). According to the MMRRC description, the homozygote phenotype is likely to be similar to the hemizygote phenotype; mice are viable and fertile, and generate almost entirely Aβ42. This strain does not carry the retinal degeneration allele Pde6b^rd1^. The 5XFAD strain is hemizygous for the amyloid precursor protein and presenilin 1 transgenes on a congenic C57BL/6 background. Given that studies on 5XFAD mice have reported a marked increase in Aβ42 [6,7] at 3 months of age followed by some cognitive impairments as early as 4 months of age [5], we chose to evaluate hemodynamic and oxygen metrics in 5XFAD mice at 3 months. We also chose this model to evaluate the impact of Aβ42 on the retina in order to reduce the number of confounding variables seen in other genetic models of AD, such as tauopathy.

**Figure 1:**
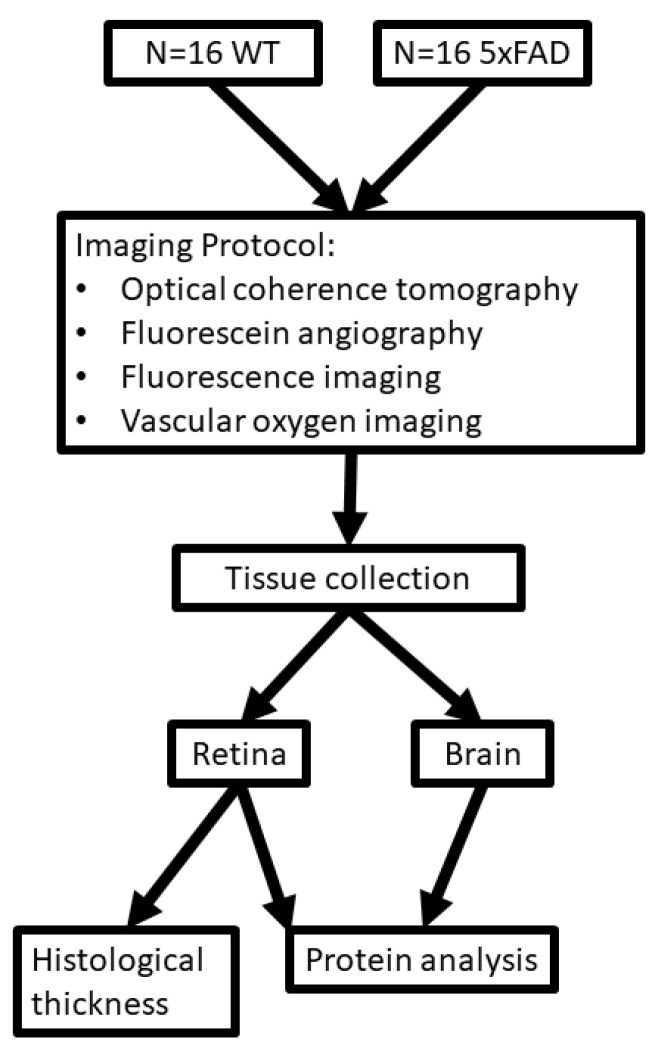
Outline for study design. Diagram showing imaging protocol, tissue collection, and protein analysis for wild-type (WT) and five-familial Alzheimer’s disease (5XFAD) groups.

One eye per mouse was selected at random for imaging. Prior to imaging, anesthesia was induced via intraperitoneal injection of ketamine (100 mg/kg) and xylazine (5 mg/kg), and the pupil was dilated. Prior to imaging, 3 mice in each group died. Spectral-domain OCT, vascular oxygen tension (PO_2_), and blood flow imaging were performed in 13 mice in each group, as described below. After euthanasia, eyes from all mice were enucleated. The imaged and fellow eyes were used for histological sectioning and protein analysis, respectively. Additionally, the brain was removed for protein analysis. The experimental outline of this study is presented in Figure 1.

### 2.2. Spectral-Domain Optical Coherence Tomography

As previously described [21,22], spectral-domain OCT imaging (Spectralis, Heidelberg Engineering, Heidelberg, Germany) was performed in two regions adjacent to the optic nerve head: nasal and temporal. Images were processed using the Heidelberg Eye Explorer software (Heidelberg Eye Explorer 1.9.10.0; Heidelberg Engineering) to measure total retinal thickness (TRT) as an average of measurements obtained in nasal and temporal regions. Due to poor image quality, TRT data were available for 12 WT and 11 5XFAD mice eyes.

### 2.3. Vascular PO_2_ and Blood Flow Imaging

For retinal vascular PO_2_ imaging, we administered fluorescent microspheres and Pd-porphine (an oxygen-sensitive molecular probe) through a femoral catheter. As previously described [21,22], a phosphorescence lifetime imaging system was used to determine PO_2_. Briefly, a frequency-domain approach was used in retinal vessels to evaluate the phosphorescence lifetime of Pd-porphine in the retinal vessels. Using the Stern-Volmer equation, PO_2_ data were derived from phosphorescence lifetime measurements. To determine O_2_ content, we summed the oxygen bound to hemoglobin and dissolved in blood. The mouse hemoglobin dissociation curve was used to calculate oxygen saturation from PO_2_ values. Arterial (O_2A_) and venous (O_2V_) oxygen contents were derived by averaging values from all vessels, and the arteriovenous oxygen content difference (O_2AV_) was calculated as O_2A_-O_2V_. As previously described [21,22], a series of images rapidly acquired at 104 Hz was used to measure blood velocity (V) in each vein based on the displacement of fluorescent microspheres over time. Blood vessel diameter (D) was determined using fluorescein angiography via injection of 10% fluorescein sodium (5 mg/kg, AK-FLUOR; Akorn, Decatur, IL, USA). In each eye, measurements of D in arteries and veins were averaged to provide mean arterial diameter (D_A_), venous diameter (D_V_), and venous velocity (V_V_), respectively. Blood flow in each vein was calculated at the product of blood V, D^2^, and π/4, and measurements were summed to calculate the total retinal blood flow (TRBF). The following equations were used to calculate DO_2_, MO_2_, and OEF: DO_2_ = TRBF × O_2A_, MO_2_ = TRBF × O_2AV_, OEF = MO_2_/DO_2_. Data were obtained from 13 WT and 13 5XFAD mice eyes.

### 2.4. Cell Layer Thickness

The eyes were sectioned to include the pupil, optic nerve, and retina, both nasally and temporally. This ensured that the sections were oriented perpendicular to the retinal surface so that measurements were not obliquely made through the retina. Using established methods, retinal sections were prepared and stained with hematoxylin and eosin (H&E). Retinal layer thickness measurements were made using a standard procedure [23]. Two digital images, nasal and temporal to the optic nerve head, were acquired and analyzed using ImageJ software (ImageJ 1.53; https://imagej.nih.gov/ij/, accessed on 13 July 2022). Layer boundaries were identified and used to calculate the thickness of the inner retina (IRL), inner plexiform layer (IPL), inner nuclear layer (INL), outer plexiform layer (OPL), outer nuclear layer (ONL), photoreceptor layer (PRL), and total retinal thickness (TRT). The thickness of the combined nerve fiber layer (NFL) and retinal ganglion cell layer (RGCL) was calculated as: NFL/RGCL = IRL − (IPL + INL). The NFL/RGCL cannot be separated in mice as in humans. Thickness data for each layer were averaged over the two regions. Due to tissue processing issues, retinal layer thickness data were available for 10 WT and 8 5XFAD mice eyes.

### 2.5. Protein Analysis

Enzyme-linked immunosorbent assays (ELISA) were performed on brain and retinal tissue extracts to quantitate the concentrations of Aβ42 and Aβ40 proteins. Briefly, mice were perfused with cold PBS, with a pH of 7.4, by intracardiac injection, followed by dissection of the retina and whole brain, which were snap-frozen in liquid nitrogen and stored at −80 °C. The retina was suspended in 70 µL of RIPA lysis buffer (Santa Cruz Biotechnology, Dallas, TX, USA), sonicated, and centrifuged at 14,000× *g* at 4 °C for 30 min. The supernatant was used for ELISA testing. Similarly, the whole brains from these mice were homogenized in RIPA lysis buffer using a tissue homogenizer and then centrifuged, and the supernatant was used as whole-cell protein extract. The protein concentration was determined using a BCA protein assay kit (23225; ThermoFisher Scientific, Waltham, MA, USA). Aβ levels in the extracts were quantified using commercial ELISA kits: Mouse Aβ40 Elisa kit (KMB3481; Invitrogen, Waltham, MA, USA) and Human Aβ42 ELISA kit (KHB3441; Invitrogen). Standard curves for Aβ40 and Aβ42 were determined in all experiments using the provided standards. Duplicate samples were used for Aβ40 and Aβ42 experiments. Due to the small volume of retinal tissue in a single eye, protein level data were available for 4 WT and 9 5XFAD mice brains and retinas.

### 2.6. Immunoreactivity for Amyloid Angiopathy

Immunofluorescence staining was performed on transverse retinal sections, as previously described [23]. Briefly, the slides were deparaffinized, boiled for 20 min in a citrate antigen retrieval buffer (10 mM sodium citrate, 0.05% Tween 20, pH 6.0), and blocked with 10% normal donkey serum. According to previous studies investigating retinal vascular amyloidosis, CD31 was used as a marker for vascular endothelium [24,25]. The retinal tissue was incubated with the primary antibodies: rat anti-CD31 (1:100; Abcam ab56299, RRID: AB_940884) and rabbit anti-Aβ42 (1:100; Cell Signaling Technology Cat# 14974, RRID: AB_2798671), and incubated with a corresponding fluorescence-conjugated secondary antibody and 4′,6-diamidino-2-phenylindole, dihydrochloride (DAPI) (nuclear marker, color blue) (Jackson ImmunoResearch, West Grove, PA, USA). No staining was observed in the imaging of the negative control: staining performed without primary antibody. The sections were visualized with a confocal microscope (LSM880, ZEISS Microscopy, Jena, Germany) using 63× magnification.

### 2.7. Statistical Analysis

No outliers were identified, and normality of the data distributions was confirmed. Unpaired, two-tailed Student’s *t*-tests were used for statistical analysis of two-group comparisons. Pearson’s correlation analysis was used to relate O_2A_ to V_V_, and O_2A_ (and O_2V_) to NFL/RGCL thickness. Statistical significance was accepted at *p* < 0.05. We used SPSS Statistics, version 24 (IBM Armonk, New York, NY, USA) for all data analyses. Statistical power analysis was performed only on measured metrics. With a sample size of 13, differences in O_2A_, O_2V_, D_V_, and V_V_ could be detected with 72%, 55%, 53%, and 98% power at the alpha level of 0.05, respectively. The study had 89% and 68% power to detect a correlation coefficient of 0.57 and 0.53 at the alpha level of 0.05 with a sample size of 26 (V_V_ and O_2A_) and 19 (O_2A_ and NFL/RGCL thickness), respectively. Power calculations were performed using G*power 3.1.9.4.

## 3. Results

### 3.1. Hemodynamic Metrics

**Figure 2:**
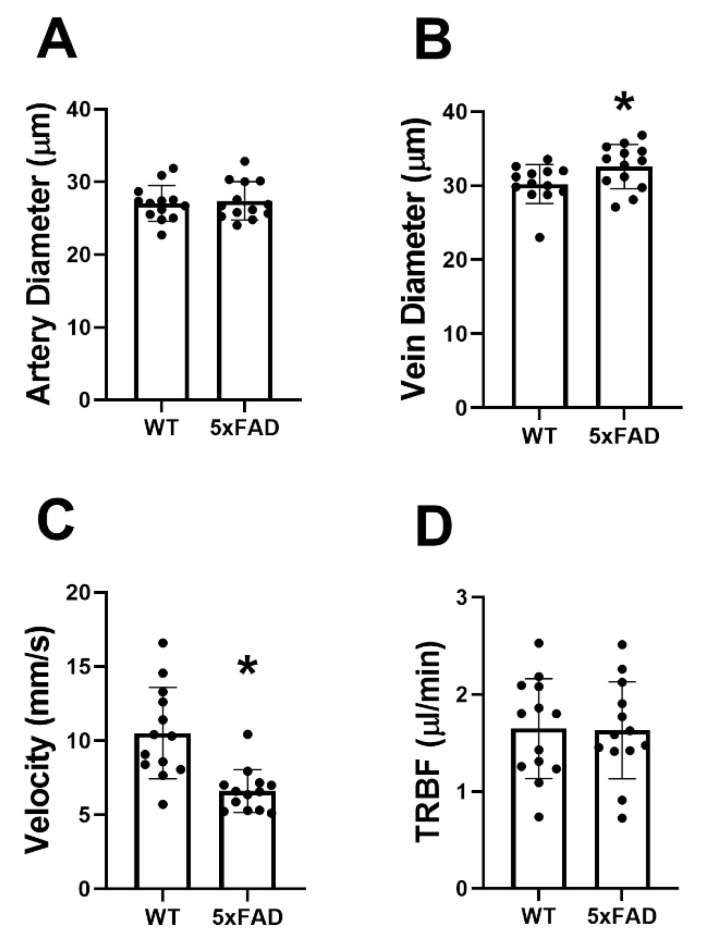
Retinal vessel diameter, venous blood velocity, and total retinal blood flow (TRBF) in wild-type (WT) and five-familial Alzheimer’s disease (5XFAD) mice. (**A**) Retinal arterial diameter, (**B**) venous diameter, (**C**) venous velocity, and (**D**) TRBF in WT and 5XFAD groups. The data are presented as mean ± SD. * *p* < 0.05.

The mean and standard deviation of D_A_, D_V_, V_V_, and TRBF for each group are shown in Figure 2. In the WT group, D_A_, D_V_, V_V_, and TRBF were 27 ± 2 μm, 30 ± 3 μm, 10.5 ± 3.1 mm/s, and 1.73 ± 0.52 /μLmin, respectively. D_V_ (33 ± 3 μm) was significantly increased, whereas V_V_ (6.6 ± 1.4 mm/s) was reduced in the 5XFAD group (*p* ≤ 0.04). No significant differences were detected in D_A_ (27 ± 3 μm) and TRBF (1.63 ± 0.50 mm/s) in the 5XFAD group compared with the WT group (*p* ≥ 0.7).

### 3.2. Oxygen Metrics

**Figure 3:**
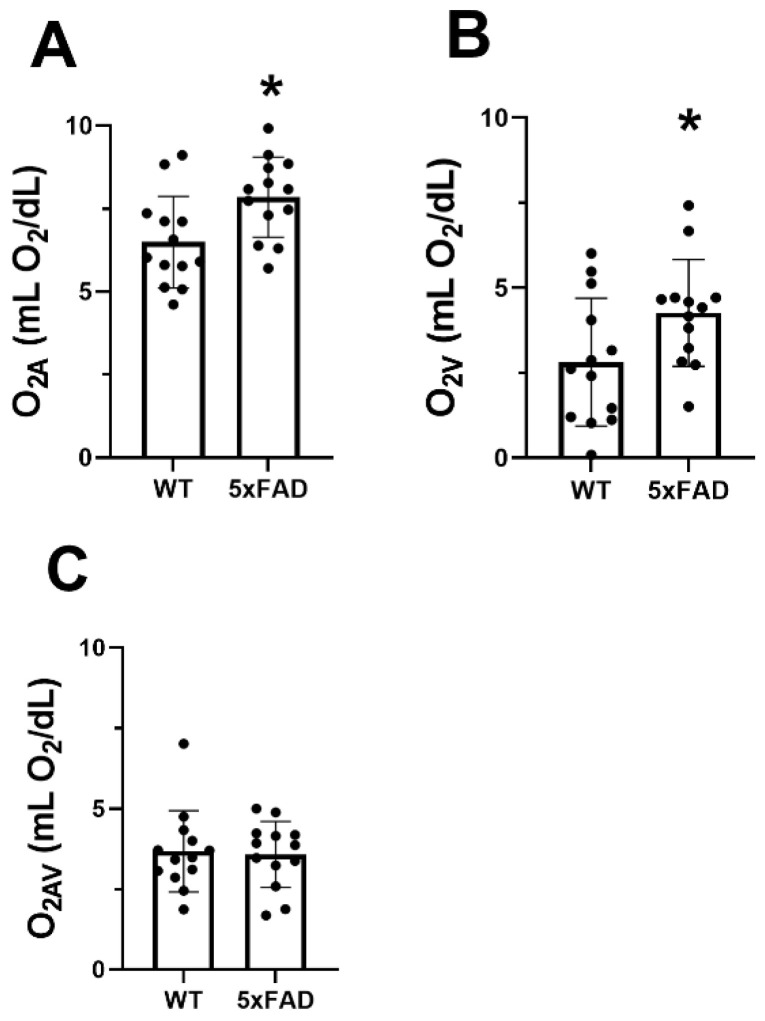
Vascular oxygen contents in wild-type (WT) and five-familial Alzheimer’s disease (5XFAD) mice. (**A**) Retinal arterial oxygen content (O_2A_), (**B**) venous oxygen content (O_2V_), and (**C**) arteriovenous oxygen content difference (O_2AV_) in WT and 5XFAD groups. The data are presented as mean ± SD. * *p* < 0.05.

The mean and standard deviation of O_2A_, O_2V_, and O_2AV_ for each group are presented in Figure 3. O_2A_, O_2V_, and O_2AV_ of the WT group were 6.5 ± 1.4, 2.8 ± 1.9, and 3.7 ± 1.3 mLO_2_/dL, respectively. O_2A_ (7.8 ± 1.2 mLO_2_/dL) and O_2V_ (4.3 ± 1.6 mLO_2_/dL) were significantly increased in the 5XFAD group (*p* ≤ 0.04). No significant difference was detected in O_2AV_ between the two groups (*p* = 0.83).

In the WT group, MO_2_, DO_2_, and OEF were 57 ± 17 nLO_2_/min, 107 ± 39 nLO_2_/min, and 0.59 ± 0.21, respectively. Compared with the WT group, no differences were detected in MO_2_ (60 ± 20 nLO_2_/min), DO_2_ (133 ± 31 nLO_2_/min), and OEF (0.46 ± 0.15) in the 5XFAD group (*p* ≥ 0.1).

### 3.3. Retinal Layer Thickness

**Figure 4:**
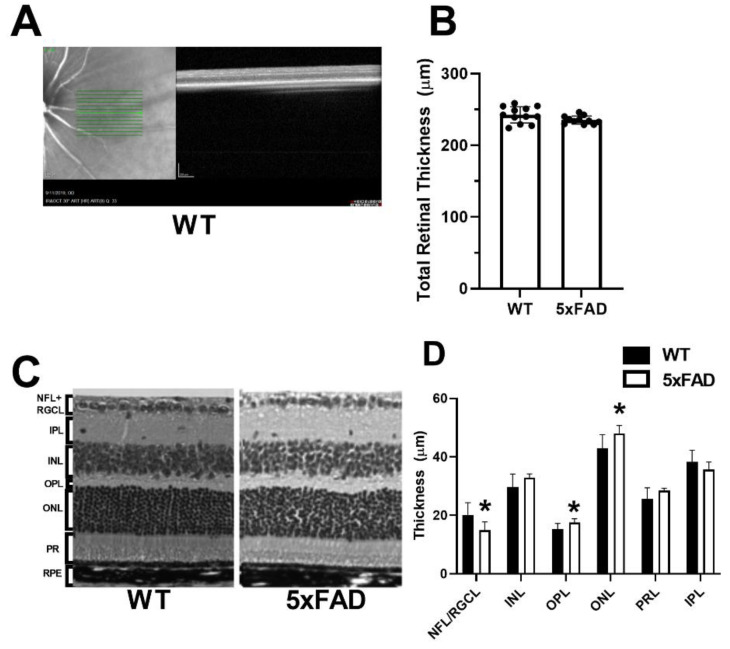
Measurements of total and retinal layer thickness in wild-type (WT) and five-familial Alzheimer’s disease (5XFAD) mice. (**A**) Region of imaging and representative optical coherence tomography image of the retina. (**B**) Total retinal thickness measured by spectral domain optical coherence tomography. (**C**) Representative retinal layer thicknesses measured from histological sections. (**D**) Thickness measurements of nerve fiber layer/retinal ganglion cell (NFL/RGCL), inner plexiform layer (IPL), inner nuclear layer (INL), outer plexiform layer (OPL), outer nuclear layer (ONL), and photoreceptor layer (PRL). The data are presented as mean ± SD. * *p* < 0.05.

Region of imaging and a representative OCT image acquired in a WT mouse are displayed in Figure 4A. The mean and standard deviation of TRT measurements, determined by OCT imaging for each group, are displayed in Figure 4B. In the WT group, TRT was 242 ± 11 µm (*N* = 12). Compared with the WT group, a marginally significant difference was detected in TRT (235 ± 6 µm) (*N* = 11) in the 5XFAD group (*p* = 0.07).

Representative H&E sections used to compare layer thickness are displayed in Figure 4C. The mean and standard deviation of retinal layer thickness measurements by histology evaluation, stratified by group, is shown in Figure 4D. In the WT group (*N* = 10), the thickness of each retinal layer was as follows: NFL/RGCL: 16.2 ± 4.2, IPL: 36.7 ± 4.0, INL: 31.0 ± 4.4, OPL: 14.6 ± 2.0, ONL: 44.3 ± 4.6, and PRL: 29.1 ± 3.9 µm. Compared with the WT group, the thickness of OPL (17.2 ± 1.3) and ONL (47.6 ± 2.7 µm) were increased, whereas the NFL/RGCL thickness (15.1 ± 2.8 µm) was decreased in the 5XFAD group (*N* = 9) (*p* ≤ 0.01). Compared with the WT group, no significant difference was detected in thickness of INL, IPL, or PR (*p* > 0.06).

### 3.4. Aβ42/40 Protein Evaluation in the Retina and Brain

**Figure 5:**
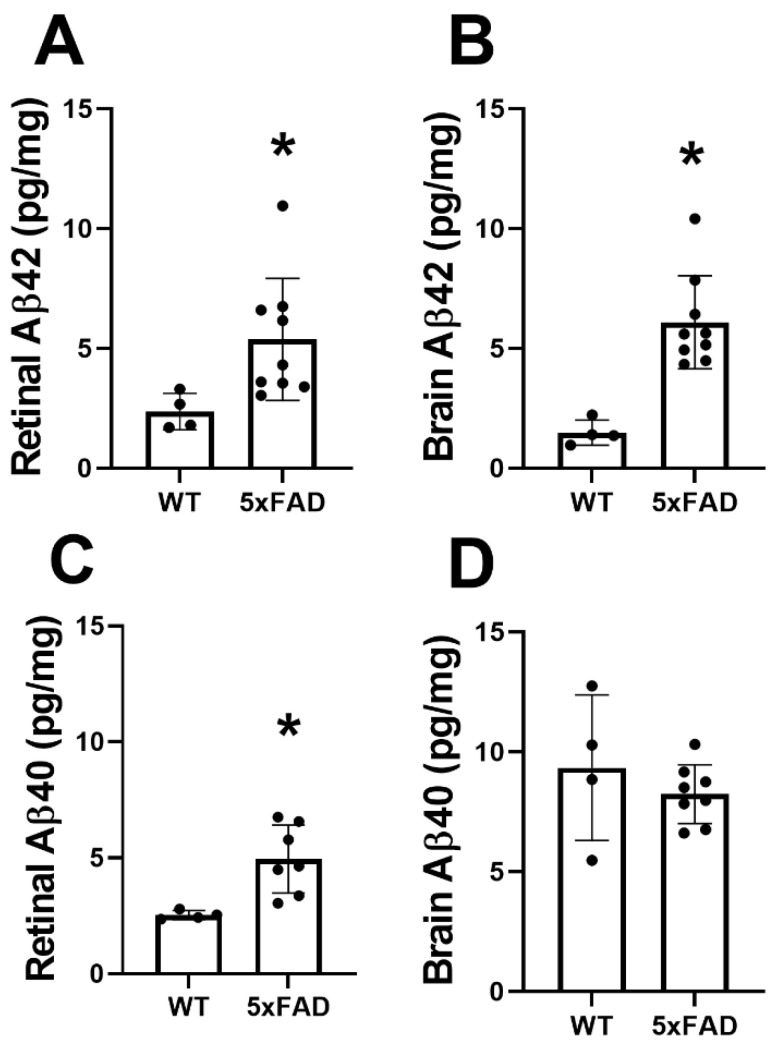
Amyloid-beta (Aβ) protein levels in retinas and brains of wild-type (WT) and five-familial Alzheimer’s disease (5XFAD) mice. Aβ42 levels in the (**A**) retina and (**B**) brain. Aβ40 levels in the (**C**) retina and (**D**) brain. The data are presented as mean ± SD. * *p* < 0.05.

The mean and standard deviation of Aβ42 and AB40 protein levels in the retina are shown in Figure 5A,C, respectively. In the WT group (*N* = 4), Aβ42 and Aβ40 were 2.4 ± 0.8 and 2.5 ± 0.2 pg/mg, respectively. Compared with the WT group, Aβ42 (5.4 ± 2.6 pg/mg) and Aβ40 (4.9 ± 1.5 pg/mg) were increased in the 5XFAD group (*N* = 9) (*p* ≤ 0.04).

The mean and standard deviation of Aβ42 and Aβ40 protein levels in the brain are shown in Figure 5B,D, respectively. In the WT group (*N* = 4), Aβ42 and Aβ40 were 1.4 ± 0.5 and 9.3 ± 3.0 pg/mg, respectively. Compared with the WT group, Aβ42 (6.1 ± 1.9 pg/mg) was increased (*p* = 0.001) in the 5XFAD group (*N* = 9), whereas no significant difference was observed in levels of Aβ40 (8.2 ± 1.2 pg/mg) (*p* = 0.4).

### 3.5. Retinal Amyloid Angiopathy

**Figure 6:**
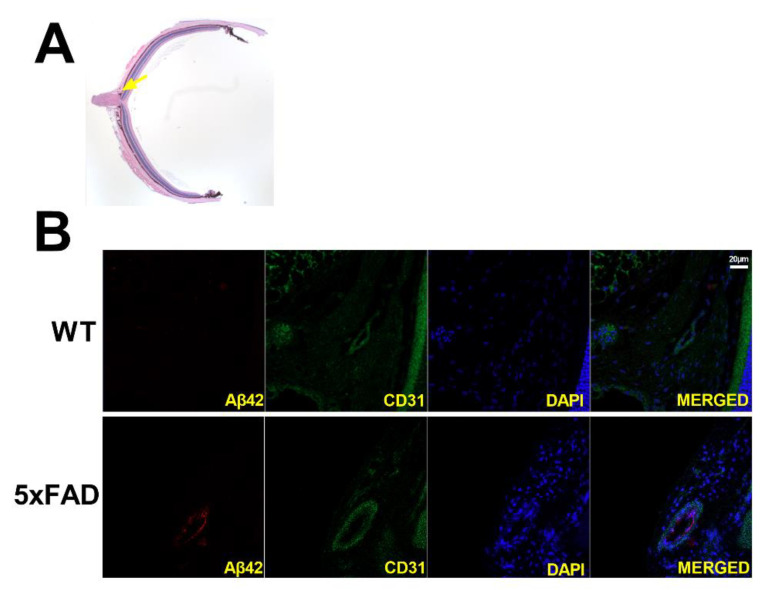
(**A**) Representative H&E-stained transverse retinal section and yellow arrow highlighting area of interest. Amyloid angiopathy is presented in transverse retinal sections of wild-type (WT) and five-familial Alzheimer’s disease (5XFAD) mice (**B**). Amyloid-beta 42 (Aβ42) is presented in red, endothelial cells in green, and nuclei in blue. Scale bar, 20 μm.

Immunofluorescence staining of retinal transverse sections from 5XFAD and WT mice was used to qualitatively evaluate amyloid angiopathy. Representative examples of retinal vessels (depicted in green), colocalized with Aβ protein (depicted in red) are shown in Figure 6. Aβ42 protein expression was present in the 5XFAD retina, whereas no staining of the Aβ42 protein was observed in the WT retina. The displayed size difference in vessels between 5XFAD and WT does not represent group differences.

### 3.6. Associations

**Figure 7:**
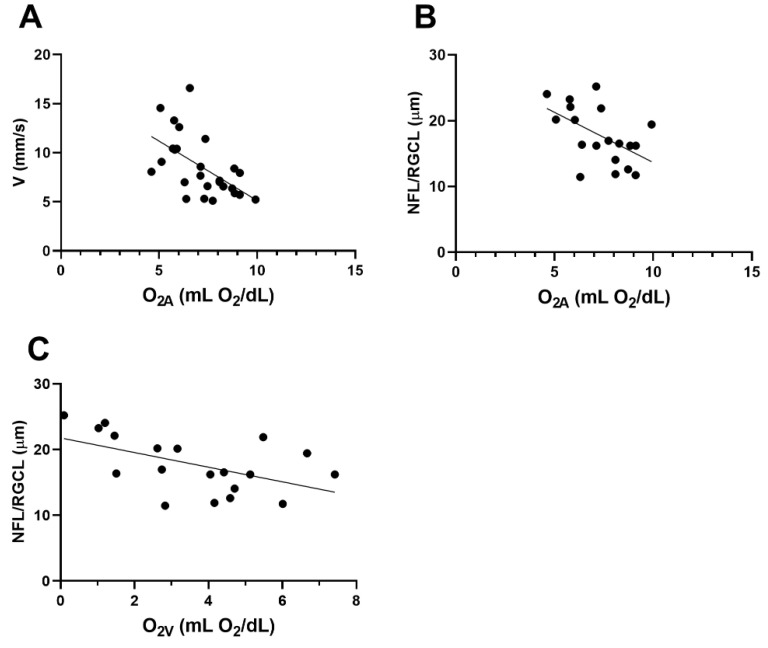
Based on compiled data from wild-type (WT) and five-familial Alzheimer’s disease (5XFAD) mice: (**A**) relationship between venous velocity (V_V_) and arterial oxygen content (O_2A_). (**B**) and (**C**) The relationships of nerve fiber layer/retinal ganglion cell (NFL/RGCL) thickness to O_2A_ and venous oxygen content (O_2V_), respectively.

The relation between O_2A_ and V_V_ is displayed in Figure 7A. Based on the compiled data from both groups, an inverse linear association between O_2A_ and V_V_ was observed (*r* = −0.57; *N* = 26; *p* = 0.002). The relationships of NFL/RGCL thickness to O_2A_ and O_2V_ are shown in Figure 7B,C, respectively. NFL/RGCL thickness was inversely related to both O_2A_ and O_2V_ (*r* = −0.53; *N* = 19; *p* = 0.02).

## 4. Discussion

Retinal vascular physiological changes in D_V_, V_V_, O_2A,_ and O_2V_ coupled with tissue anatomical alterations in OPL, ONL, PRL, and NFL/RGCL thickness were detected in 5XFAD mice at 3 months of age, concurrent with an increase in Aβ42 levels in both the retina and brain. With several novel findings, we confirmed our hypothesis that alterations in retinal vessel caliber, velocity, and oxygen content were present in 5XFAD mice; however, we did not detect changes in TRBF, MO_2_, DO_2_, or OEF.

The first major finding was that D_V_ increased, whereas V_V_ decreased, in the 5XFAD mice at 3 months of age. In AD patients, cerebral amyloid angiopathy is characterized by the deposition of Aβ in the walls of cerebral and leptomeningeal vessels and results in the abnormal distribution of blood flow due to the thickening of vessel walls and consequent narrowing of the lumen [26]. Studies have shown that collagen and Aβ accumulate in capillaries [27,28] and veins [29,30] in postmortem AD brains [31]. In an evaluation of postmortem retinas, correlations were found between retinal vascular (major retinal vessels and capillaries) abnormalities (Aβ plaques and pericyte loss) and cerebral Aβ plaques, cerebral amyloid angiopathy, and clinical status [25]. Consistent with our findings, reduced retinal venous blood velocity was observed in AD patients with cerebral and retinal amyloid angiopathy [15,16,17]. Moreover, reported pericyte loss in capillaries of postmortem AD retinas [25] and capillary degeneration in a mouse model of AD [24] likely affect blood flow regulation. Therefore, we suggest that increased Aβ42 deposition and possibly collagen-thickened vein vessel walls resulted in the subsequent narrowing of the lumina and decreased pliability of vessels, reducing their capacity to normally dilate or contract. These pathophysiological changes may have contributed to an autoregulatory increase in D_V_ and decrease in V_V_ to maintain TRBF.

The second major finding was that O_2A_ and O_2V_ were increased in the 5XFAD mice at 3 months of age. Similarly, others have reported an increase in retinal oxygen saturation in arterioles and venules in AD patients [13,14]. Given that AD results in amyloid angiopathy, the thickening of vessel walls may lead to a decrease in diffusion of glucose and oxygen from the circulatory system, including the retina. For example, oxygen saturation in the retina was increased in patients with diabetic retinopathy, attributed in part to the thickening of capillary vessel walls, which increases the distance oxygen must travel to reach its destination [32]. The observed thinning of NFL/RGCL in the current study also supports reduced oxygen utilization in this layer. These factors would tend to increase O_2V_ but not O_2A_, and O_2AV_ and MO_2_ were not found to differ in the two groups of mice. Additional investigation is needed to fully understand these vascular oxygen content findings.

The third major finding was that no statistically significant changes in MO_2_, DO_2_, and OEF were observed in 5XFAD mice at 3 months of age. Given the evidence for retinal amyloid angiopathy and degeneration of RGCL in AD, it is expected that these will become abnormal at some point. For instance, using fluorodeoxyglucose positron emission tomography (FDG-PET), a method that measures glucose metabolism, previous studies have found consistent patterns of brain metabolic dysfunction that occur prior to cell death in AD patients [33,34]. In the 5XFAD mouse model, FDG-PET has detected reduced cerebral metabolic rates by 13 months [35]. Given that the NFL/RGCL make up a small part (approximately 19%) of the inner retina, MO_2_ was likely reduced to some extent but did not reach significance. Additionally, because we found no difference in TRBF and an increase in O_2A_, DO_2_ should have mathematically been increased in the 5XFAD group, but due to the variability in our data, we did not detect a difference in DO_2_. In sum, 3 months may have been too early to detect impairments in MO_2_, DO_2_, and OEF.

The fourth major finding was reduced NFL/RGCL thickness and increased OPL, ONL, and PRL thickness, coupled with no significant change in TRT in the 5XFAD mice. Past research has reported thinning of RGCL in AD patients, whereas thickening was observed in the ONL and PRL, using ultrahigh-resolution OCT [36]. Others have reported RGCL loss in patients [37,38], as well as in other AD animal models, such as APP-PS1ΔE9 [39] and 3xTg-AD mouse models [40]. Although our understanding of the molecular mechanisms for inner retinal thinning remains incomplete, studies have shown that the activation of the JNK pathway, a stress modulator that is activated by Aβ, plays a part in the degeneration of neurons in the brain and retina [41]. Using an inhibitor of JNK, D-JNKI1—in a phase III clinical trial for treating intraocular inflammation resulting from surgery or trauma [42]—Buccarello et al. reported that monthly intraperitoneal injections of D-JNKI1 preserved the RGCL in a mouse model of AD [41]. Given that patients also tolerate this drug, it may have a significant translational impact in treating AD. If oxygen diffusion from retinal circulation is decreased, outer and inner retinal tissue may be hypoxic to different degrees, so the ONL/PRL thickening and NFL/RGCL thinning could in part be due to cytotoxic edema and cell death, respectively. We propose three possible contributors to this: (1) disproportionate deposition of amyloid in the innermost retina and in and along vessels, (2) suboptimal regulation of TRBF to fully compensate for impeded oxygen diffusion that appears to result in a slight reduction in MO_2_ (this is based on thinning in the NFL/RGCL), and (3) altered distribution of blood flow such that there are greater reductions in flow in the superficial than the deep capillary plexus [43]. These factors affect the NFL/RGCL more severely, which accounts for cell loss; however, there is still swelling found in the OPL and ONL. Further research is needed to understand the pathophysiology of NFL/RGCL thinning and OPL/ONL/PRL thickening. In the current study, TRT was not significantly different between AD and WT mice. However, TRT measured by histology was lower than measurements obtained in vivo by OCT imaging. These differences in TRT measurements may be attributed to histological processing causing shrinkage in tissue or software limitations that are not adjusting for differences in optical properties between mouse and human models.

The fifth major finding was that the expression of Aβ42 was increased in both the brain and retina at 3 months of age in the 5XFAD mice, consistent with previous studies of both the retina [6,7] and brain [6,44]. The dominant pathogenesis theory of AD suggests that the production and clearance of Aβ42 and related Aβ peptides is most likely the initiator of AD disease and its downstream physiological effects [45]. We are the first to report increased Aβ42 levels, coupled with changes in V_V_, O_2A_, O_2V_, and retinal cell layers in the 5XFAD mice. To date, there is a dearth of non-invasive biomarkers for AD. Ocular imaging that detects physiological and anatomical abnormalities associated with AD onset may become an essential modality for early diagnosis. Typically, Aβ42 is deposited in senile plaques, whereas Aβ40 normally deposits in the vascular wall of cerebral amyloid angiopathy (CAA) [46]. Previous literature has reported that CAA, in which plaques are increased in cerebral blood vessels and tissue, was observed in more than 80% of AD patients [47]. Moreover, Aβ deposits in the brain and retina of AD patients were associated with accumulated deposits within retinal vasculature [25,48]. However, further research is needed to understand the link between Aβ42 and its impact on V_V_, O_2A_, and retinal thickness.

A sixth major finding of the current study was the association of retinal vascular oxygen content with blood velocity and retinal layer thickness. There was a significant inverse relationship between O_2A_ and V_V_ in the retina. One possible explanation for this finding is the presence of amyloid angiopathy and thickening of the vessel walls due to the deposition of Aβ. Under physiological conditions, elevated O_2A_ triggers a regulatory response that leads to vasoconstriction [49] and reduced TRBF [50]. However, under pathological conditions as in 5XFAD, the observed increase in O_2A_ may be attributed to reduced oxygen diffusion across thickened vessel walls upstream of the measured site, in vessels traveling to the eye or even farther along toward the heart. If inner retinal vessels are thickened, we would anticipate a decrease in oxygen diffusion that could result in hypoxic conditions, supported by the increased D_V_ observed in vascular regulation. In addition to oxygen regulation, retinal vessel caliber can be affected by VEGF levels. Blair et al. found a significant increase in D_V_ in response to exogenous VEGF administration [51]. Although VEGF levels were not measured in the current study, the literature has shown a marked increase in plasma levels of VEGF in AD patients [52]. This vasodilatory response is counteracted by a reduction in V_V_ in order to maintain blood flow. Nevertheless, if hypoxia is prolonged, cells may ultimately undergo death as evidenced by our finding of decreased NFL/RGCL thickness and elevated O_2V,_ although O_2AV_ showed no significant change.

There were several limitations in the current study. First, these results may not be generalizable to other mice models of AD or human AD, as there may be variations in disease progression and phenotype according to genetic models, species, sex, and age. Future studies are warranted to determine the effect of sex and age on retinal hemodynamics and oxygen metrics in WT and 5XFAD mice. Second, factors such as the constants used in the Stern-Volmer equation may be different within the retinal tissue environment; moreover, hemoglobin concentrations, the oxygen–hemoglobin dissociation curve, and blood pH were not measured. However, given that the same values were used in both groups, these inaccuracies likely did not impact the relative changes observed in the 5XFAD mouse model compared with WT. Third, WT mice were not littermates to the 5XFAD mice, and six WT mice purchased from Charles River Laboratories were not screened for retinal degeneration mutations. Therefore, genetic variances may have contributed to differences in the WT group as well as between groups. Fourth, given the small volume of retinal tissue from a single eye, protein analysis was limited such that other proteins such as amyloid precursor protein could not be evaluated. Finally, small sample size may have limited our ability to detect some differences (type-II error) between groups and correlations between parameters.

## 5. Conclusions

Concurrent alterations in retinal vascular physiology, anatomy, and tissue Aβ42 protein levels in 5XFAD mice at 3 months of age corresponded to previously reported findings in human AD. Overall, these findings suggest that this mouse model can be utilized for studying the pathophysiology of AD and evaluating potential therapies.

## Data Availability

All data generated during this study have been included in the manuscript. Further data supporting the findings of this study are available from the corresponding authors on request.

## Abbreviations

Aβ	beta-amyloid
AD	Alzheimer’s disease
CAA	cerebral amyloid angiopathy
D_A_	arterial diameter
D_V_	venous diameter
DO_2_	oxygen delivery
ELISA	Enzyme-linked immunosorbent assays
5XFAD	five-familial Alzheimer’s disease
FDG-PET	fluorodeoxyglucose positron emission tomography
INL	inner nuclear layer
IPL	inner plexiform layer
NFL/RGCL	nerve fiber layer/retinal ganglion cell layer
MO_2_	oxygen metabolism
O_2A_	arterial oxygen content
O_2AV_	arteriovenous oxygen content difference
O_2V_	venous oxygen content
OCT	optical coherence tomography
OEF	oxygen extraction fraction
ONL	outer nuclear layer
OPL	outer plexiform layer
PO_2_	vascular oxygen tension
PRL	photoreceptor layer
RNFL	retinal nerve fiber layer
TRBF	total retinal blood flow
TRT	total retinal thickness
V	venous blood velocity
WT	wild type
